# Experimental and Numerical Insights into the Semi-Circular Bend (SCB) Test for Tensile Strength Estimation in Rock-like Materials

**DOI:** 10.3390/ma18184285

**Published:** 2025-09-12

**Authors:** Rashid Hajivand Dastgerdi, Agnieszka A. Malinowska

**Affiliations:** Faculty of Geo-Data Science, Geodesy, and Environmental Engineering, AGH University of Krakow, 30-059 Kraków, Poland

**Keywords:** SCB test, tensile strength, sandstone, FEM, brittle fracture

## Abstract

The uncracked semi-circular bend (SCB) test has recently gained attention as a simple and material-efficient method for determining the tensile strength of brittle geomaterials. However, as reported in the literature and confirmed by our experiments, localized damage at the roller supports remains a critical limitation that may compromise measurement accuracy and test validity. This study addresses this limitation through experimental testing on red and gray sandstone, complemented by numerical simulations to provide deeper insight into stress distribution and fracture mechanisms in the SCB test. Experimental results showed that six out of twelve specimens experienced local damage, ranging from slight crushing and surficial cracking at the base roller zones in red sandstone to rock chipping in gray sandstone. The stiffer sandstone exhibited more severe local damage due to its limited deformability. These damages were attributed to minor geometric imperfections introduced during sample preparation. Nevertheless, all tests yielded valid tensile strength values, with SCB results showing good agreement with Brazilian test outcomes and demonstrating significantly lower coefficients of variation. Finite element simulations confirmed that crack initiation consistently occurred at the middle of the flat edge under pure tensile stress, indicating a mode I fracture mechanism. Numerical analyses further revealed pronounced stress concentrations, particularly compressive stresses, at the roller contact zones, induced by the specimen’s low span-to-depth ratio, which increased the fracture load required for failure.

## 1. Introduction

**Figure 1:**
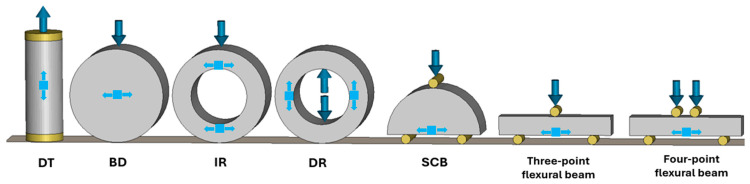
Common and recently developed test methods for determining the tensile strength of geomaterials.

Tensile strength is a fundamental property governing crack initiation and failure in brittle geomaterials such as rock and concrete. Accurate determination of this property is essential for reliable design and failure prediction in civil, petroleum, and mining applications. While direct tensile testing theoretically offers the most accurate measurement of this property, they are often limited by challenges related to specimen gripping, alignment, and premature failure at contact points. Such challenges reduce the repeatability and reliability of the results. Consequently, indirect methods are commonly used to estimate tensile strength, including the Brazilian disk test, flexural beam tests, and ring tests, which induce tensile failure through compressive or flexural loading. The Brazilian disk test (also known as splitting test or Brazilian tensile strength test), while simple and widely applied, induces a biaxial stress state rather than pure tension, which often leads to non-uniform crack initiation and failure away from the specimen center, thereby complicating interpretation [1]. The ring test generates tensile stresses concentrated along the inner circumference, making results highly sensitive to local flaws, while also requiring difficult specimen preparation and precise loading alignment [2]. Flexural beam tests, though conceptually straightforward, demand relatively large specimens. These drawbacks highlight the need for alternative testing methods that combine experimental simplicity with reliable stress states for brittle materials. The semi-circular bend (SCB) test has emerged as a practical alternative for evaluating the fracture toughness and tensile strength of brittle geomaterials due to its simple geometry, minimal material requirement, and flexibility in both static and dynamic conditions [3]. In particular, the uncracked SCB configuration, in which a semi-circular specimen without a notch is subjected to three-point bending, has been used to estimate tensile strength based on peak load and geometric factors. Representative examples of these established techniques are illustrated in Figure 1.

In recent years, various researchers have successfully employed SCB methods for mechanical characterization across various material types. For example, Yu et al. [4] used cracked and uncracked SCB specimens with a Split Hopkinson Pressure Bar to measure both the dynamic tensile strength and fracture toughness of sandstone, showing that the SCB method is effective for high strain rate testing. Elghazel et al. [5] conducted both cracked and uncracked SCB tests on calcium phosphate ceramics to evaluate their tensile strength and fracture toughness. Aliha et al. [6] applied both cracked and uncracked semi-circular bend (SCB) tests to evaluate the tensile strength and mode I fracture toughness of polymer concrete and conventional concrete. Choi et al. [7] compared the uncracked SCB and Brazilian tests for tensile strength determination of Hwangdeung granite and Boryung sandstone, employing Aliha’s [8] formulation to estimate BTS from SCB results, and reported comparable tensile strength values between the two methods. Bahrami and Ayatollahi [9] experimentally and numerically demonstrated that in cracked SCB testing, frictional contact at the roller supports can induce localized damage and alter stress distributions, thereby compromising the accuracy of the measured fracture toughness. More recently, Aliha et al. [10] investigated asphalt mixtures and observed that some uncracked SCB specimens failed prematurely due to localized damage around the lower support rollers. This premature failure was attributed to material heterogeneity and grain misalignment, which generated stress concentrations along the grain–matrix boundaries in those regions.

The present study focuses on the local damage induced in uncracked SCB specimens, a limitation that has not yet been comprehensively addressed in the literature. To this end, experimental tests were conducted on red and gray sandstone, complemented by numerical simulations to provide deeper insight into stress distribution and fracture mechanisms in the uncracked SCB test.

## 2. SCB Test

**Figure 2:**
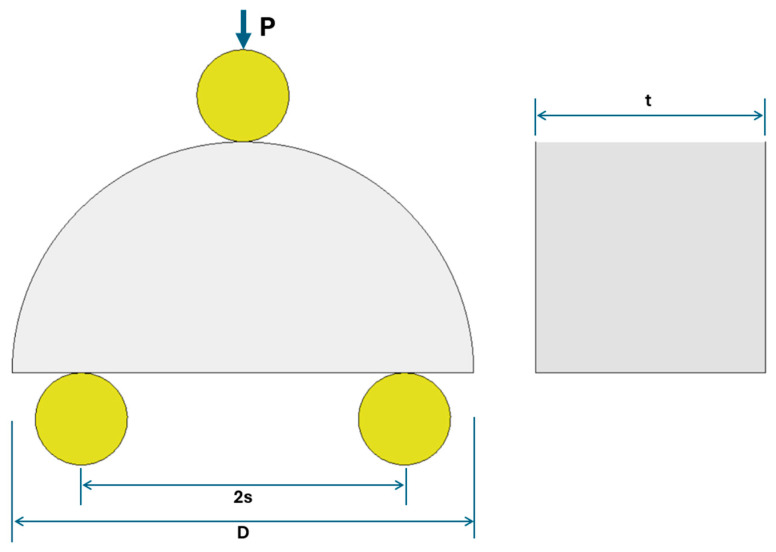
SCB test geometry and loading configuration.

The semi-circular bend (SCB) test is a well-established technique for evaluating the tensile strength and fracture behavior of brittle materials such as rock and concrete. It is a modified three-point bending test in which a vertical load is applied to a semi-circular specimen, inducing failure at the middle of the flat edge through a mode I tensile cracking mechanism. While initially developed for fracture toughness assessment, it was subsequently adapted for indirect tensile strength evaluation using unnotched specimens [11]. The standard specimen consists of a half-disk with diameter D and thickness t, supported on two bottom rollers spaced at a distance S, and loaded vertically at the midpoint of the upper edge. The specimen geometry and loading setup are illustrated in Figure 2.

For estimating tensile strength from uncracked SCB test results, Aliha [8] proposed a modified formula derived from three-dimensional linear-elastic finite element analyses in ABAQUS. A systematic parametric study was conducted for thickness-to-diameter ratios (t/D) ranging from 0.1 to 0.5 and span-to-diameter ratios (S/D) ranging from 0.5 to 0.8. The formulation was then validated against experimental results on Harsin marble, a homogeneous and isotropic rock, and was shown to provide tensile strength values consistent with those obtained from the Brazilian tensile strength test [8]. Based on this study, the tensile strength of SCB specimens can be calculated using the following expressions:

(1) $$\sigma_{t}^{SCB}=K_{t}^{SCB}\cdot C\frac{2P}{\pi tD}$$

(2) $$K_{t}^{SCB}=0.146\left(\frac{t}{D}\right)+0.8896$$

(3) $$C=4.02\left(\frac{S}{D}\right)+1.052$$

where P represents the peak load at failure, and $K_{t}^{SCB}$ and C are geometry-related correction factors. These expressions have been successfully employed in previous studies on sandstone and other geomaterials [4,5,6,7,9,10] and are applied in the following sections to determine the tensile strength of uncracked SCB sandstone specimens.

## 3. Brazilian Splitting Test

**Figure 3:**
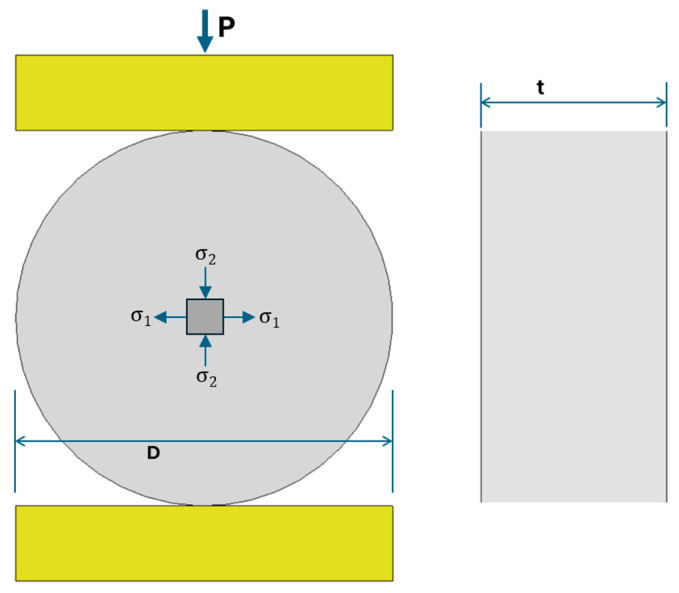
Schematic of the Brazilian test showing diametral loading and internal stress distribution.

The Brazilian splitting test (BTS) is the ISRM-recommended method and the most widely used indirect approach for determining the tensile strength of brittle geomaterials. In this method, a disk-shaped specimen is loaded diametrically in compression, producing a biaxial stress state at the disk center with vertical compression and horizontal tension, as illustrated in Figure 3. The horizontal tensile stress, generated by lateral expansion through the Poisson effect and internal stress redistribution, eventually causes tensile failure. Despite its limitations such as stress concentrations at the loading platens, the induction of a biaxial rather than a pure tensile stress state, and the potential for non-uniform crack initiation or off-center fracture, the BTS remains the standard reference method against which alternative techniques are evaluated [1,12,13].

As the applied load increases, the horizontal tensile stress within the central region intensifies and eventually exceeds the material’s tensile strength. The tensile strength ($\sigma_{t}$) of Brazilian disk specimens is commonly determined using the classical two-dimensional elasticity solution proposed by Mellor and Hawkes [14]:

(4) $$\sigma_{t}^{BTS}=\frac{2P}{\pi tD}$$

Here, P denotes the applied diametral load, while t and D represent the specimen thickness and diameter, respectively. Because the BTS configuration is inherently subjected to a three-dimensional stress state, reliance on a purely two-dimensional solution may lead to inaccuracies. To overcome this limitation, Aliha performed three-dimensional finite element analyses and introduced a thickness correction factor ($K_{t}^{BTS}$), thereby extending the classical expression to a generalized three-dimensional formulation [8]. The tensile strength of Brazilian disk specimens can therefore be determined using the following expression:

(5) $$\sigma_{t}^{BTS}=K_{t}^{BTS}\cdot \frac{2P}{\pi tD}$$

(6) $$K_{t}^{BTS}=0.312\left(\frac{t}{D}\right)+0.964$$

In this study, both equations are employed to calculate the Brazilian tensile strength of sandstone, allowing for a clearer comparison between the two formulations in the following sections.

## 4. Laboratory Tests

Red and gray sandstones were selected as the rock materials for this study. Their mechanical properties were evaluated through uniaxial compressive strength (UCS) and Brazilian tensile strength (BTS) tests, and the principal physical and mechanical characteristics are summarized in Table 1.

**Figure 4:**
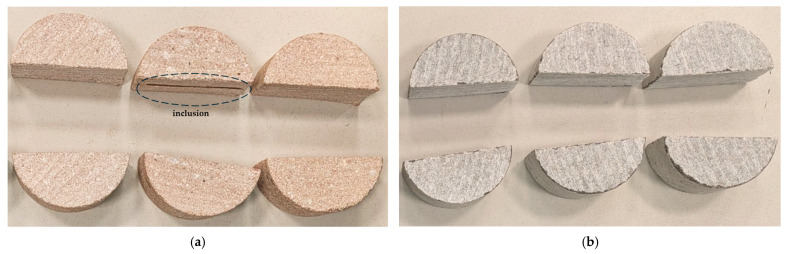
Prepared SCB specimens of (**a**) red sandstone and (**b**) gray sandstone.

For the SCB tests, cylindrical sandstone cores with a diameter of 63 mm were cut into 31 mm thick disks and then halved to create semi-circular specimens, as illustrated in Figure 4. The types and number of tests performed are summarized in Table 2.

**Figure 5:**
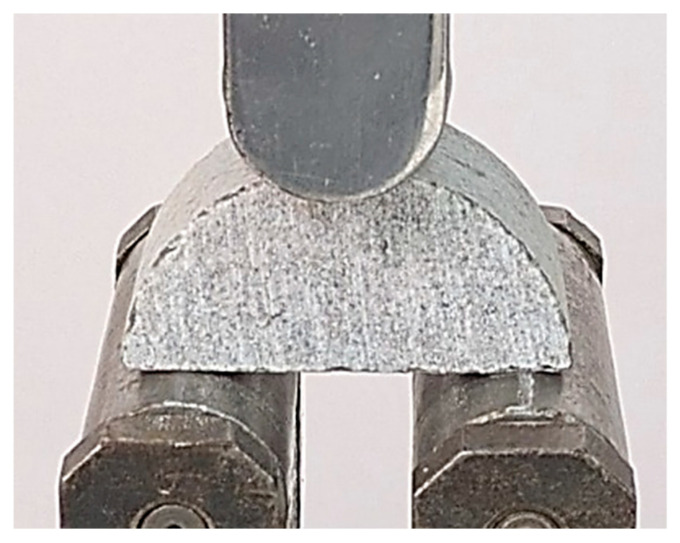
Gray sandstone SCB specimen positioned in the three-point bending test setup.

SCB specimens were tested under three-point bending using a servo-controlled universal testing machine. The load was applied vertically at the midpoint of the specimen’s upper surface, while two bottom rollers provided support. Each roller had a diameter of 30 mm, and the span between supports was fixed at 47 mm for all tests. Loading was applied under displacement control at a constant rate of 2 mm/min. Special attention was given to specimen alignment to minimize unintended bending or torsional effects during testing. Figure 5 shows the SCB specimen positioned between the rollers prior to testing.

## 5. Numerical Simulations

**Figure 6:**
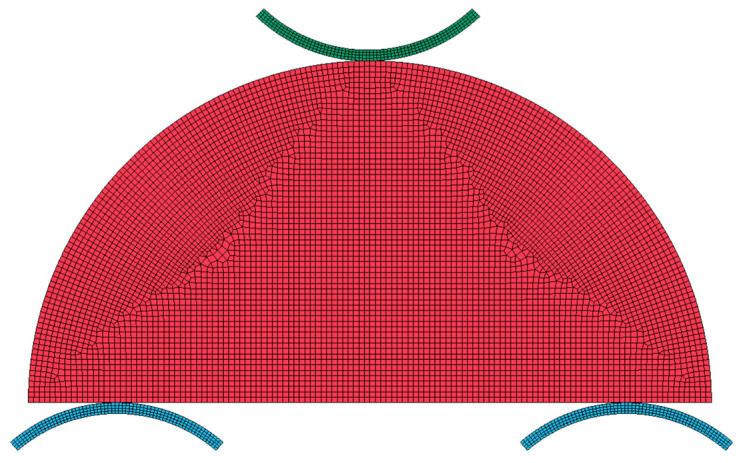
SCB model mesh.

Numerical simulations of the SCB test were conducted using the commercial finite element software ANSYS/LS-DYNA (2025 R1 (4.12.0))to investigate failure mode, damage evolution, and stress distribution under three-point bending. A two-dimensional plane strain approach was adopted, replicating the experimental geometry with a disk diameter of 63 mm and a roller span of 47 mm. In conventional FEM, cracks often propagate along element boundaries, which can introduce mesh bias and produce unrealistic fracture trajectories. To overcome this, mesh size and element-type sensitivity analyses were performed to determine the minimum mesh density required for convergence and the most suitable element family for fracture modeling. The final model consisted of 6664 quadrilateral and triangular elements with a maximum element size of 0.5 mm, as shown in Figure 6.

A three-point bending setup was modeled using rigid steel rollers with standard structural steel properties. The lower rollers were fixed, while a vertical downward displacement was applied through the upper roller. Contact between the sandstone specimen and the rollers was defined using the CONTACT_2D_AUTOMATIC_NODE_TO _SURFACE keyword with default parameters for rock–steel interaction. Sandstone behavior was captured using the MAT_RHT material model. The Riedel–Hiermaier–Thoma (RHT) model, originally developed for concrete, accounts for brittle fracture, pressure-dependent strength, and post-peak degradation through three distinct stress surfaces: elastic yield, failure, and residual strength [15]. The RHT formulation has been successfully applied to a range of brittle geomaterials, including dynamic failure in sandstone [16], tensile and compressive behavior of granite [17], and rock fracture and damage evolution under impact loading [18]. These applications confirm RHT model capability for simulating failure and damage in concrete-like materials, justifying its use here for modeling sandstone under SCB loading. The average mechanical properties of red sandstone, listed in Table 1, were used as input for the RHT model. Fracture development was simulated using element erosion technique. For this purpose, the MAT_ADD_EROSION keyword was selected with a maximum principal strain (MXEPS) of 0.15, allowing elements exceeding this threshold to be deleted, thereby realistically capturing material failure and crack propagation. This value was calibrated based on sensitivity analysis, as it yielded the closest agreement with the experimental SCB results. Due to the small time steps inherent in explicit dynamic simulations, a time-scaling technique was employed to reduce computational time. The total simulation duration was set to 1 s, ensuring quasi-static loading and minimizing strain rate effects.

## 6. Results and Discussion

### 6.1. Experimental Observations

**Figure 7:**
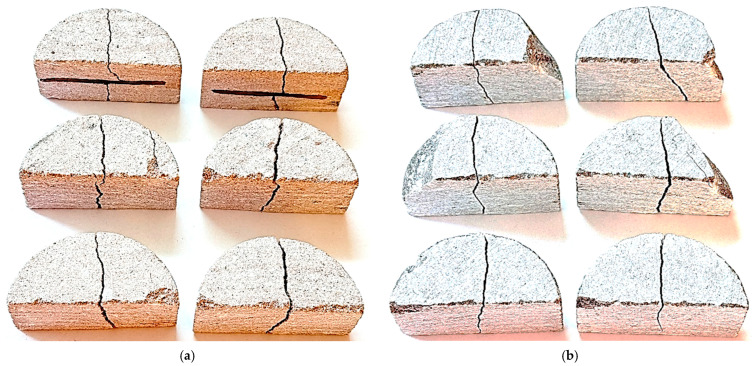
Fracture patterns of red (**a**) and gray (**b**) sandstone specimens obtained from SCB tests.

As illustrated in Figure 7a, the red sandstone specimens exhibited nearly mid-span vertical fractures. Cracks initiated in the tensile zone beneath the loading line and propagated toward the specimen edge, occasionally deviating due to local stress concentrations. This behavior aligns with previous reports on other brittle materials under bending, where cracks typically initiate in tension and propagate along preferred planes [19,20]. Minor local damage and superficial cracking were also observed in two specimens near the roller–specimen contact zones, likely resulting from slight material distortions introduced during sample preparation. In addition, two specimens contained mid-depth inclusions that caused local crack deflection along the inclusion–sandstone interface before penetrating through the inclusion; however, the corresponding fracture loads remained comparable to those of the inclusion-free specimens, indicating only a minor influence on strength. For gray sandstone, the fracture pattern was likewise predominantly observed at the mid-span, as shown in Figure 7b. However, four out of six specimens exhibited chipping near the base roller supports, attributed to geometric imperfections introduced during sample preparation. Despite this localized damage, the intact portions of the specimens continued to carry load between the rollers until final tensile failure. The fracture loads recorded for the chipped specimens were comparable to those of the undamaged ones, confirming the reliability of the SCB test results for gray sandstone. These observations indicate that in distorted samples, stiffer sandstone is more sensitive to geometric imperfections, leading to chipping and localized damage, whereas softer sandstone can deform more readily under load, reducing stress concentrations and sustaining less damage.

**Figure 8:**
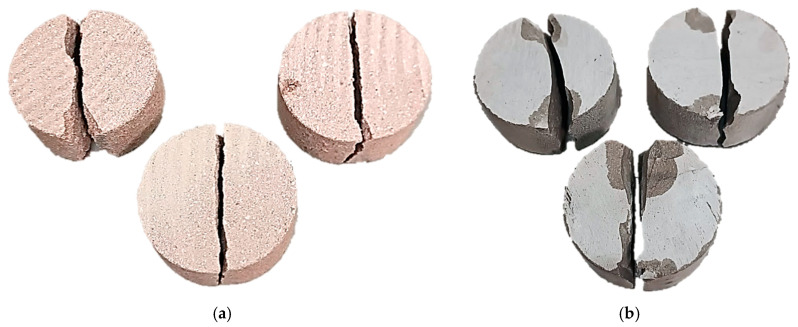
Typical failure patterns of red (**a**) and gray (**b**) sandstone specimens obtained from Brazilian splitting tests.

Typical failure patterns of red and gray sandstone under Brazilian splitting tests are presented in Figure 8.

Table 3 presents the tensile strength results obtained from both SCB and Brazilian splitting tests. Tensile strength values from the SCB test were determined using the formulation proposed by Aliha [8], whereas the Brazilian splitting test results were evaluated using both the classical formula [14] and the Aliha [8] formula. For red sandstone, the SCB test produced an average strength of 2.67 MPa, which closely matched the Brazilian results of 2.60 MPa from Hawks and 2.86 MPa from Aliha formula, while also exhibiting lower variability with a coefficient of variation of 16% compared to 30%. For gray sandstone, the SCB test yielded an average tensile strength of 5.36 MPa with a coefficient of variation of only 6%, in contrast to 5.92 MPa from Hawks and 6.63 MPa from Aliha formula obtained by the Brazilian test, both of which showed a much higher coefficient of variation of 42%. These results confirm that the SCB method provides tensile strength values consistent with those of the Brazilian test, but with significantly improved repeatability. Moreover, the Aliha [8] formula systematically predicted higher tensile strength values for the Brazilian test than the classical relation for both rock types.

### 6.2. Numerical Observations

**Figure 9:**
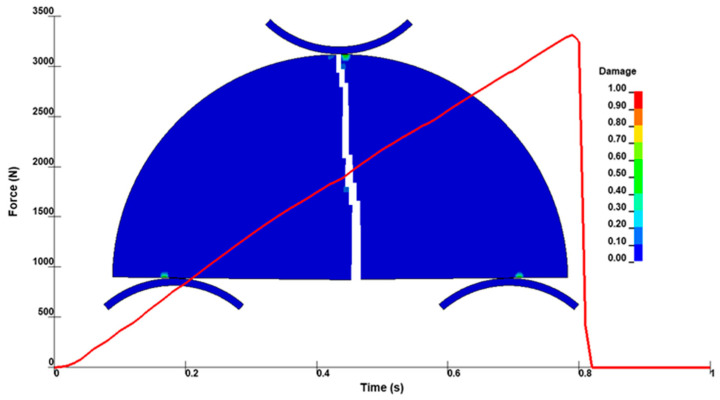
Fracture and damage evolution with the load–time response in the red sandstone FEM model.

Figure 9 presents the fracture and damage evolution in the red sandstone SCB model, along with its corresponding load–time response. The FEM simulation predicted a peak fracture load of 3331 N, which is in close agreement with the experimental average of 3350 N, corresponding to a deviation of only 0.6% and thereby confirming the accuracy and reliability of the numerical model for further investigations. Moreover, localized damage near the roller contact areas is evident in the simulation. In the FEM model, fully damaged elements (damage value = 1) were removed using an element erosion technique to simulate material separation and crack formation, while partially damaged elements remained active. Although the crack initiated slightly away from the mid-span, stress redistribution redirected it toward the center, where it subsequently propagated vertically through the SCB specimen.

**Figure 10:**
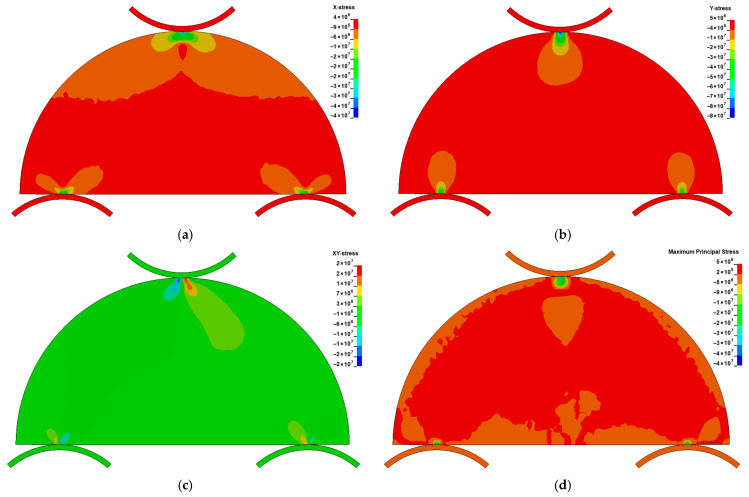
Stress components in the SCB specimen at the onset of failure. (**a**) horizontal stress (**b**) vertical stress (**c**) shear stress (**d**) maximum principal stress.

In Figure 10, the horizontal stress ($S_{x}$), vertical stress ($S_{y}$), shear stress ($S_{xy}$), and maximum principal stress at the onset of failure in the SCB test on red sandstone are shown. Pronounced stress concentrations are evident at the roller–specimen contact zones in the SCB test.

**Figure 11:**
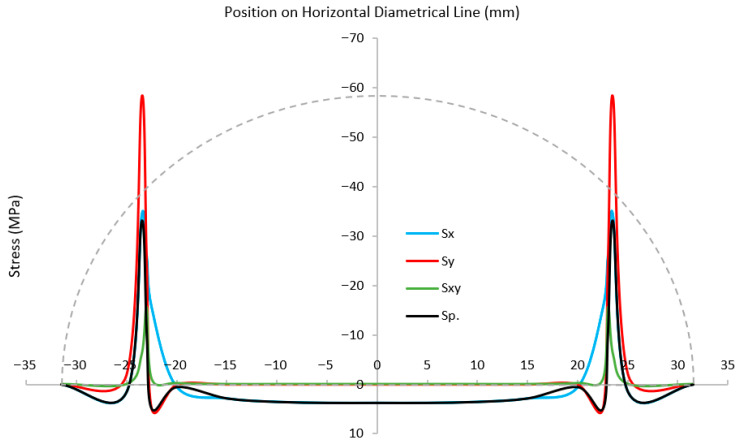
Stress distribution along the horizontal line in the SCB specimen.

**Figure 12:**
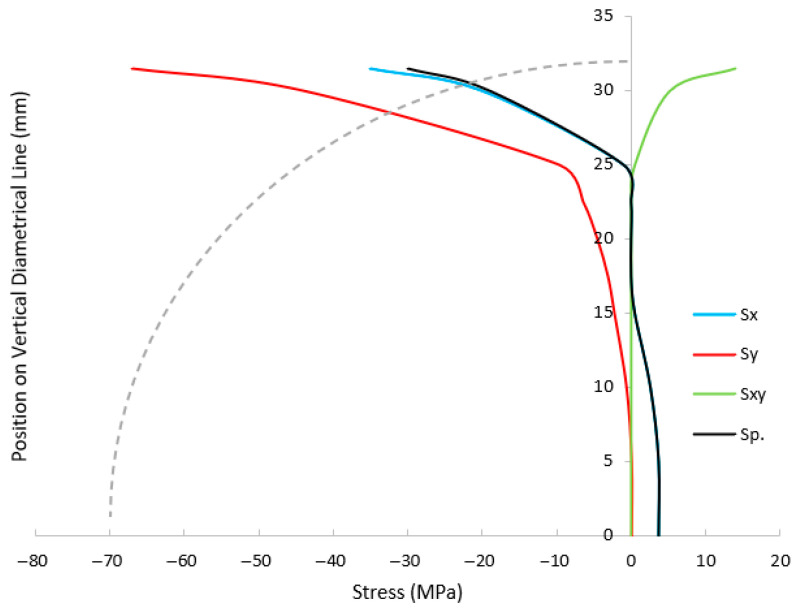
Stress distribution along the vertical line in the SCB specimen.

Figure 11 and Figure 12 show the stress components along horizontal and vertical lines in the SCB specimen at the onset of failure. High compressive stress concentrations are observed near the roller contact zones, where the applied load is transferred into the specimen. Due to the SCB’s low span-to-depth ratio, a relatively high load is required to induce fracture, which intensifies stress at the supports [21,22]. Geometric distortions, particularly along the flat edge, reduce the effective contact area between the rollers and the specimen, thereby amplifying stress concentrations at the support zones. These elevated stresses are the primary cause of the observed local damage. In red sandstone, which is a softer rock, slight deformation of the material helps to relieve these concentrations, mitigating the influence of geometric imperfections. In addition, any loading misalignment that reduces roller–specimen contact can further intensify stress concentrations and lead to localized damage in these zones.

At the mid-span of the flat edge, the horizontal and maximum principal stresses converge, while vertical and shear stresses are negligible, indicating a pure tensile stress state. This zone corresponds to the crack initiation point, consistent with tensile failure mechanisms in brittle materials. Although the local tensile stress slightly exceeds the input tensile strength in the RHT model, the simulated failure mode aligns well with expected rock fracture behavior.

## 7. Conclusions

This study evaluated the uncracked semi-circular bend (SCB) test for determining the tensile strength of brittle geomaterials, with particular emphasis on the problem of roller-induced local damage that has not been comprehensively addressed in previous research. Experimental testing on red and gray sandstone demonstrated that six out of twelve specimens developed roller-related damage. In red sandstone, this appeared mainly as surficial cracking and minor crushing at the roller supports, while in gray sandstone, chipping was observed due to its higher stiffness and limited ability to redistribute stresses. These localized damages were linked to minor geometric imperfections introduced during sample preparation. Reduced roller–specimen contact caused by geometric distortions or loading misalignment amplifies stress concentrations at the supports, leading to local damage. Despite this, all specimens produced valid tensile strength values, and SCB results showed close agreement with Brazilian splitting test (BTS) outcomes, while exhibiting substantially lower coefficients of variation. For red sandstone, variability decreased from 30% in BTS to 16% in SCB, and for gray sandstone, from 42% to 6%. This confirms the improved repeatability and reliability of the SCB method compared with BTS. Finite element simulations validated the experimental findings and provided deeper insight into the fracture process. The analyses confirmed that cracks were consistently initiated at the mid-span of the flat edge under pure tensile stress, indicating a mode I fracture mechanism. Strong compressive stress concentrations were also observed at the roller–specimen interfaces, caused by the low span-to-depth ratio, which artificially increased the fracture load required for failure. These insights underline the mechanical origin of roller-induced damage and explain why stiffer rocks are more prone to such effects. Overall, the results confirm that the uncracked SCB test is a reliable and material-efficient alternative to the BTS for measuring tensile strength in red and gray sandstone. However, high precision in specimen preparation is essential, especially for high-strength rocks, where roller-induced damage is more severe.

## Figures and Tables

**Table 1:** Material properties of the red and gray sandstone.

Mechanical Properties	Unit	Red Sandstone	Gray Sandstone
Density (*ρ*)	Kg/m^3^	2363	2687
Average Young’s Modulus in Compression (*E*)	GPa	6.1	8.5
Average Poisson’s ratio (*ν*)	-	0.18	0.2
Average Uniaxial Compressive Strength (UCS)	MPa	36.3	53
Average Brazilian Tensile Strength (BTS)	MPa	2.60	5.9

**Table 2:** Performed tests on red sandstone.

Test	Red Sandstone			Gray Sandstone		
	UCS	BTS	SCB	UCS	BTS	SCB
Number of samples	8	5	6	8	5	6

**Table 3:** Tensile strength values obtained from SCB and Brazilian tests.

	SCB Fracture Load (N)	SCB Tensile Strength (Pa)	SCB Average Tensile Strength (MPa)	BTS Average Tensile Strength (MPa) [Equation (4)]	BTS Average Tensile Strength (MPa) [Equation (5)]
Red Sandstone	3740	2.99	2.67 ± 0.43 (COV = 16%)	2.60 ± 0.78 (COV = 30%)	2.86 ± 0.85 (COV = 30%)
	4200	3.35			
	3300	2.63			
	3000	2.39			
	2700	2.15			
	3200	2.5			
Gray Sandstone	6280	5.02	5.36 ± 0.33 (COV = 6%)	5.92 ± 2.49 (COV = 42%)	6.63 ± 2.79 (COV = 42%)
	6550	5.23			
	6750	5.39			
	7500	5.99			
	6500	5.19			
	6700	5.35			

## Data Availability

The original contributions presented in this study are included in the article. Further inquiries can be directed to the corresponding author.
